# “Exploring the Link Between Oral Lichen Planus and Xerostomia: A Systematic Literature Review”

**DOI:** 10.1002/iid3.70101

**Published:** 2024-12-19

**Authors:** Farzaneh AghaHosseini, Maryam Tahmasebinasab, Mehdi Vatanpour

**Affiliations:** ^1^ Dental Research Center, Dentistry Research Institute Tehran University of Medical Sciences Tehran Iran; ^2^ Department of Oral Medicine, Faculty of Dentistry Tehran University of Medical Sciences Tehran Iran; ^3^ The Academy of Medical Sciences Tehran Tehran Iran; ^4^ Department of Oral Medicine, School of Dentistry Tehran University of Medical Sciences Tehran Iran; ^5^ Department of Endodontics, School of Dentistry Islamic Azad University Tehran Iran

**Keywords:** autoimmune disorder, dry mouth, etiopathogenesis, hyposalivation, oral lichen planus, xerostomia

## Abstract

**Introduction:**

Oral lichen planus (OLP) is a chronic disorder affecting the oral mucosa, potentially associated with xerostomia, either independently or concurrently. Research suggests that approximately 45% of patients with erythematous and ulcerative OLP may experience dry mouth sensations. The aim of this systematic review is to assess the current literature regarding the potential relationship or co‐occurrence of xerostomia with OLP. Understanding this association is imperative for the development of comprehensive management strategies and the improvement of patient outcomes.

**Method and Material:**

The study followed the PRISMA 2020 checklist and included human studies, specifically investigating xerostomia in patients with OLP. After screening 897 articles, 9 studies were selected based on predefined criteria Quality assessment was conducted using the Cochrane risk of bias tools: ROB 2 for RCTs and ROBINS‐I for non‐randomized studies Scale was conducted to evaluate potential biases in study design, selection, and outcomes.

**Result:**

A systematic review of nine studies (1960–2023) examining xerostomia in OLP patients found a significant reduction in unstimulated salivary flow rates in many cases. Although evidence links xerostomia with OLP, a definitive causal relationship remains unestablished. Some studies highlighted Candida infection, altered saliva protein expression, and inflammation‐related nerve damage as contributing factors to dry mouth in OLP patients.

**Discussion and Conclusion:**

This systematic review examines the potential relationship between OLP and xerostomia, focusing on factors such as salivary flow, histopathological changes, and immune‐related mechanisms. While some studies suggest a link between OLP and reduced saliva production, no definitive causal relationship has been established. The review identified significant research gaps, including inconsistent methodologies and a lack of standardized criteria. Future studies should explore different OLP forms, receptor interactions, immune responses, and neuropeptides to gain a better understanding of xerostomia's etiopathogenesis and improve management strategies for OLP patients.

## 1 Introduction

Oral lichen planus (OLP) is a chronic oral mucosal disease classified as an oral potentially malignant disorder (OPMD) with a prevalence of 1%–2% in the adult population [32]. It is characterized by a T‐cell‐mediated inflammatory response, where a band‐like infiltrate of T‐cells accumulates beneath apoptotic epithelial cells and a disrupted basement membrane [33]. OLP presents with a range of intraoral manifestations, including reticular, plaque‐like, papular, atrophic/erosive, ulcerative, and bullous types [34]. These clinical subtypes not only vary in presentation but also in their associated symptoms and impact on patient's quality of life [35]. Forty‐five percent of patients with erythematous and ulcerative forms of OLP also experience xerostomia, which is characterized by a subjective sensation of dry mouth and the presence of highly viscous saliva [36, 37]. Several studies have explored the association of OLP with xerostomia, a condition marked by a subjective sensation of dry mouth and often accompanied by reduced salivary flow [38, 39, 40, 41]. However, these studies are limited by variations in methodologies, and insufficient consideration of confounding factors, which hinder a comprehensive understanding of relationship. Moreover, the potential role of xerostomia in exacerbating OLP symptoms and its impact on the disease's progression remain unclear. Future research should adopt a more integrated approach, incorporating immunological, biochemical, and clinical data to better understand the complex between OLP and xerostomia. This systematic review has provided a comprehensive overview of the existing literature on the potential relationship or co‐occurrence of OLP and xerostomia.

## 2 Materials and Methods

This systematic review adheres to the PRISMA guidelines and is registered in the PROSPERO database under the code CRD42023426014. The primary research question of this review is: “What is the potential relationship or co‐occurrence of xerostomia with OLP in terms of factors, such as reduced salivary flow, histopathological changes, and etiopathogenesis?”

The components of the PICO framework include:

Population (P): Individuals diagnosed with Oral Lichen Planus (OLP).

Intervention (I): Assessment of xerostomia (dry mouth sensation), including factors such as reduced salivary flow, histopathological changes, and etiopathogenesis.

Comparison (C): Patients with OLP but no xerostomia, or healthy controls.

Outcomes (O): Potential relationship or co‐occurrence of xerostomia with OLP. Here, please remember the specified PICO questions:

1. Comparison of histopathological changes in patients with OPL and xerostomia with the control group.
2. Comparison of salivary flow changes in patients with OPL and xerostomia with the control group.
3. Comparison of the ethiopathogenesis in patients with OPL and xerostomia with the control group.

A comprehensive search in electronic databases, including MEDLINE/PubMed, Scopus, Web of Science (ISI), Embase, and Google Scholar, covering studies published from 1960 to the present. MeSH terms, Emtree terms, and free‐text keywords were used. The search strategy combined the following terms: (“Oral Lichen Planus” OR “Lichen Planus, Oral”) AND (xerostomia OR Hyposalivation OR “Dry Mouth” OR Asialia OR “Mouth Dryness”). The selection process involved the following steps:

1. Establishing inclusion and exclusion criteria.
2. Reviewing titles of all retrieved articles.
3. Evaluating abstracts of relevant articles.
4. Assessing full‐text articles for eligibility.
5. Appraising the quality of selected studies using predefined criteria.
6. Evaluating the risk of bias and heterogeneity.
7. Selecting suitable methods for data synthesis and analysis.

Inclusion Criteria

– Studies conducted on human subjects with OLP.
– Studies specifically addressing xerostomia or dry mouth in OLP patients.
– Randomized controlled trials (RCTs), non‐randomized controlled trials.
– Articles available in full text.

Exclusion Criteria

– Studies focusing on other oral mucosal conditions.
– Studies not including xerostomia or dry mouth as a primary or secondary outcome.
– Review papers, case reports, conference papers, letters, monographs, and unpublished data.

The data from the included studies were independently extracted by two authors (Farzaneh AghaHosseini and Maryam Tahmasebinasab), encompassing study design, sample size, participant characteristics, intervention details, comparison groups, and outcomes. Quality assessment was conducted using the Cochrane risk of bias tools: ROB 2 for RCTs and ROBINS‐I for non‐randomized studies. Each study was assessed for risk of bias and categorized as low, moderate, or high risk based on the Cochrane tools. The overall quality of evidence was evaluated using the GRADE approach, where applicable, to provide a comprehensive assessment of the findings. Due to methodological heterogeneity among the included studies, conducting a meta‐analysis was not viable. Instead, a systematic review table was utilized to summarize the findings (Table 1).

**Table 1:** Studies performed on xerostomia or dry mouth.

	Author/year	Aim of study	Type of study	Method	Conclusion
1.	Gandara et al. [9]	Studying the histochemistry of full saliva and saliva secreted from parotid and minor salivary glands	Cross sectional All: 50(25–25)	1. Examining fully stimulated and non‐stimulated saliva 2. Exploring the rate of saliva and concentration of immunoglobulin A and g plus albumin and lysosome, amylase, lactoferrin, and total protein as well as sodium, potassium, calcium, chloride, and phosphorus in stimulated saliva secreted from parotid and labial salivary glands	Insignificant rate of saliva and concentration of electrolytes and their proteins
2.	Colquhoun and Ferguson [10]	Studying the relationship between OLP and xerostomia	Cross sectional All: 464 (116–348)	Questionnaire	Significance of mean xerostomia score
3.	Bokor‐Bratic, Jankovic and Dragnic [3]	Determining the prevalence of oral fungal flora in OLP patients	Cross sectional All: 180(90–90)	Analysis of d non‐stimulated saliva specimen and swab specimen for candida evaluation.	1. Significance of candida prevalence in OLP patients. 2. Lower rate of non‐stimulated saliva
4.	Artico, et al. [4]	Studying the prevalence of candida and xerostomia and hyposalivation	Cross sectional All: 98 OLP lesions (38 patients) non‐OLP lesions (28 patients) healthy subjects (32 subjects)	1. Examining candida swab from the buccal mucosa and dorsal surface of the tongue 2. Examining xerostomia with a questionnaire 3. Exploring non‐stimulated saliva rate	1. Insignificance of xerostomia in those with and without oral lesions. 2. Insignificance of prevalence of candida.
5.	Farzaneh Agha‐Hosseini, Mirzaii‐Dizgah and Mohammadpour [14]	Comparing the level of M3 receptors in non‐stimulated and stimulated saliva	Cross sectional All: 80 (40–40)	Examining M3 receptor in stimulated and non‐stimulated saliva using ELISA kit	1. Less significance of M3R in non‐stimulated saliva. 2. Less significance of M3R in stimulated saliva. 3. Higher significance of severity of xerostomia according to questionnaire
6.	Larsen, et al. [2]	Evaluating the degree of xerostomia and examining full non‐stimulated and stimulated saliva along with saliva flow rate secreted from parotid and slgA protein concentration among OLP patients and lichenoid reaction as well as oral stomatitis	Cross sectional All: 134 49=olp 19=oll 11=stomatitis 29=control	1. Evaluating xerostomia with a questionnaire 2. Evaluating stimulated and non‐stimulated saliva	1. Higher significance of xerostomia. 2. Insignificance of xerostomia with salivary secretion rate. 3. Higher significance of such concentration. 4. Lower significance of protein level in non‐stimulated saliva 5. Significantly higher protein level in stimulated saliva
7.	Agha‐Hossein, et al. [1]	Examining the flow of non‐stimulated and stimulated saliva plus the level of B5 mucin in non‐stimulated and stimulated saliva as well as serum of OLP patients.	Cross sectional All: 90 (45–45)	Examining MUC5B in stimulated and non‐stimulated saliva	1. Less significance of non‐stimulated saliva flow in OLP patients. 2. Less significance of MUC5B level in non‐stimulated saliva in OLP patients 3. Significantly higher MUC5B level in the serum of OLP patients.
8.	Tvarijonaviciute, et al. [5]	1. Examining the antioxidant status in full saliva of OLP patients and its possible relationship with the pain and draining and xerostomia 2. Exploring the changes in the antioxidant status of full saliva during treatment	Randomized, double‐blind, parallel‐group study All: 110 OLP: 55 Treatment with *Chamaemelum nobile*: 26 treatment with placebo: 29	Collection of non‐stimulated saliva on the first day of study and 4 weeks thereafter to evaluate the amount of antioxidant status in full saliva	1. Significance of salivary total antioxidant status. 2. Association between salivary total antioxidant status and increased pain and xerostomia as well as reduced draining.
9.	Agha‐Hosseini, et al. [15]	Comparing the level of M3 receptor in non‐stimulated and stimulated saliva as well as lower lip minor salivary glands	Cross sectional All: 62(40–22)	Examining M3 receptor in stimulated and non‐stimulated saliva as well as lower lip minor salivary glands using ELISA	1. Lower significance of stimulated and non‐stimulated saliva flow. 2. Significantly higher score of xerostomia severity inventory. 3. Significantly higher levels of M3 muscarinic receptors in the minor salivary glands of OLP patients.

## 3 Results

**Figure 1:**
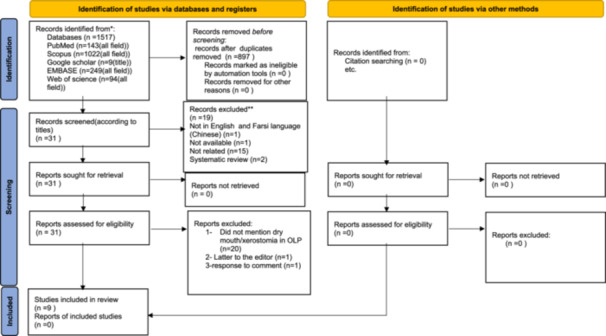
Flow diagram based on PRISMA 2020, which included searches of databases, registers, and other sources.

The studies were conducted between 1960 and 2023, using various designs including RCT and non‐RCT studies. Upon the initial search, 1517 articles were found. Subsequently, 897 articles remained after removing duplicates. Thrity‐one articles were selected based on evaluating their titles and abstracts. Ultimately, nine studies were included in the qualitative analysis [42, 43, 44, 45, 46, 47, 48, 49, 50]. Although we performed citation searching using the included studies, no additional relevant records were identified through this method (Figure 1.). After refining the initial articles, one article was randomized controlled trials (RCTs) [51] and nine articles were non‐randomized RCTs [52, 53, 54, 55, 56, 57, 58, 59], proceeding to the data extraction phase. These studies aimed to compare. Detailed information for each study is provided in Table 2. All the reviewed studies focused on xerostomia in OLP patients. The common site of OLP had not been mentioned in any of the studies. Three studies were done in Iran [60], one in New Zealand [61], Serbia [62], Denmark [63], Brazil [64], Spain [65], and the USA [66]. The mean age had not been mentioned in two studies [67, 68]. The results of four examined studies showed that in OLP patients, the non‐stimulated salivary flow rate had a significant reduction compared to the control group [69]. In two papers, the non‐stimulated saliva did not differ significantly from the control group [70, 71], and in three papers, the non‐stimulated saliva had not been examined [72, 73, 74, 88]. Two papers showed a significant association between stimulated salivary flow rate in OLP patients [74, 75, 88].

**Table 2:** List of excluded studies and the reasons for their exclusion.

1	Author	Year	Exclusion articles from this study due to ….
2.	Lundström, et al. [85]	1982	This study was excluded as it focused solely on histopathology.
3.	Oikarinen, et al. [16]	1995	… The purpose of this study was to evaluate changes in salivary variables during treatment with oral isotretinoin in patients receiving the drug for 3 months for cutaneous acne As a result, it does not match our systematic review in terms of content.
4.	Fluixa, et al. [7]	2000	… The article was in non‐English language, so it was excluded from the study.
5.	Colquhoun [8]	2002	The full text of the article was not available.
6.	Kho, et al. [17]	2013	… This article did not mention dry mouth in OLP and BMS patients.
7.	Agha‐Hosseini and Moosavi [18]	2013	This review article was excluded based on the exclusion criteria.
8.	Tanasiewicz, Hildebrandt, and Obersztyn [19]	2016	… This Review Article presents the etiopathogenesis, symptomatology, evaluation, and treatment of mouth dryness. It does not mention the effect of lichen planus on dry mouth.
9.	Frydrych [20]	2016	… This Review Article presents the etiopathogenesis, symptomatology, evaluation, and treatment of mouth dryness. It does not mention the effect of lichen planus on dry mouth.
10.	Tvarijonaviciute, et al. [21]	2017	… The fact that in terms of content, they did not give an opinion about the dry mouth of the patients and only examined the antioxidants in them.
11.	Mansourian, et al. [22]	2017	… This article did not mention dry mouth in OLP.
12.	Al‐Janaby, et al. [86]	2017	The aim of this study was to treat Oral Lichen Planus (OLP), not to address xerostomia or its treatment.
13.	Moosavi and Barati [23]	2018	… The fact that the paper was a letter to the editor.
14.	Frydrych [24]	2018	… The fact that the paper was a response to a comment.
15.	Edens, et al. [87]	2018	The aim of this study was to treat Oral Lichen Planus (OLP), not to address xerostomia or its treatment.
16.	Mansourian, et al. [25]	2018	… This article did not mention dry mouth in OLP.
17.	Agha‐Hossein, et al. [26]	2018	
18.	Mirzaii‐Dizgah, Rohani, and Mirzaii‐Dizgah [27]	2021	
19.	Sheikhbahaei and Gholizadeh [28]	2021	
20.	Yiemstan, Kridapong and Piboonratanakit [29]	2020	
21.	Osipoff, et al. [30]	2020	
22.	Çevik‐Aras, et al. [31]	2023	

Two studies analyzed the existence of Candida infection in OLP patients [76, 77]. Also, some studies investigated the expression of receptors and special proteins in saliva and showed differences between OLP and control patients [78, 79, 80, 81].

**Figure 2:**
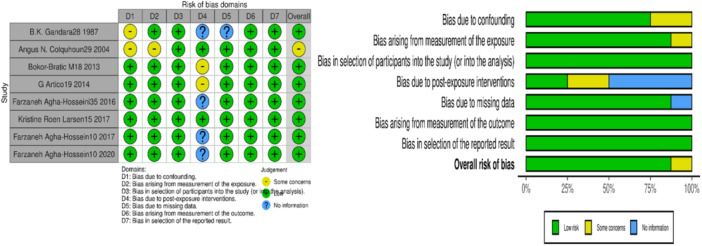
ROBINS‐I diagram shows a comprehensive summary of bias assessment. It includes the review authors' critical evaluations of the methodological quality in non‐randomized studies for each study included in the review.

In assessing the risk of bias, the articles used the Cochrane risk of bias assessment tools, specifically ROB and ROBINS‐I. Based on the ROBINS‐I analysis, All articles were categorized as low‐risk [43, 44, 45, 46, 47, 48, 49, 50]. (Figure 2) Additionally, the ROB analysis for an RCT article indicated a low risk [42]; however, since there was only one article, it was not possible to create a diagram for it. The quality level of all articles was high.

In the studies, we reviewed the potential relationship or co‐occurrence of xerostomia with OLP in relation to factors, such as reduced salivary flow, histopathological changes, and etiopathogenesis. A significant number of patients experience reduced saliva production and changes in the salivary glands. Although there is evidence of a connection between OLP and dry mouth, the studies did not definitively establish a cause‐and‐effect relationship. Some studies suggested that Candida infection and changes in saliva protein expression could contribute to dry mouth in OLP patients. Additionally, chronic inflammation‐induced nerve damage also contributes to the development of dry mouth in OLP patients Table 3.

**Table 3:** Categorization of studies performed on xerostomia or dry mouth.

No.	Author/year	Categorization of studies performed on xerostomia or dry mouth		
1	Gandara, et al. [9]		Investigating salivary function and saliva histochemistry	
2	Colquhoun and Ferguson [10]	Without quantitative and qualitative investigation of saliva	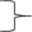	Questionnaire investigation in patients with OLP suffering from dry mouth
	Larsen, et al. [2]	Together with the quantitative and qualitative investigation of saliva		
3	Bokor‐Bratic, Jankovic, and Dragnic [3]	Without questionnaire investigation and nonstimulated quantity	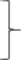	Determining the fungal microflora prevalence
	Artico, et al. [4]	With questionnaire investigation and stimulated and nonstimulated saliva quantity		
4	Tvarijonaviciute, et al. [5]	Determining total antioxidant status in the full saliva of OLP patients and its association with xerostomia		
5	Agha‐Hosseini, Mirzaii‐Dizgah, and Mohammadpour [14]	In non‐stimulated and stimulated saliva	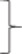	Determining cholinergic/muscarinic M3 receptors
	Agha‐Hosseini, et al. [15]	In non‐stimulated and stimulated saliva as well as lip minor salivary glands		
6	Agha‐Hosseini, et al. [1]	Determining the level of B5 mucin in non‐stimulated and stimulated saliva as well as serum of OLP patients with xerostomia		

## 4 Discussion

In this systematic review, the aim was to investigate the potential relationship or co‐occurrence of xerostomia with OLP in relation to factors, such as reduced salivary flow, histopathological changes, and etiopathogenesis. No evidence was available for PICO question 1, so this study focused on comparing factors related to xerostomia in OLP.

The initial studies (Table 2) focused on examining the histochemistry of whole saliva as well as saliva produced by the parotid and minor salivary glands. The findings indicated no variations in the volume of unstimulated saliva produced [9]. With a mean value of 0.41 ± 0.05 mL/min, which was within the normal range for the study population. However, these findings should be interpreted cautiously, as no standardized cut‐off values for xerostomia were employed, and age‐related salivary flow reduction was not adequately accounted for, despite OLP predominantly affecting individuals over 50 years old. Another study from 2004 assessed xerostomia using the Xerostomia Inventory questionnaire. All participants reported experiencing dry mouth symptoms, but the study did not quantify the severity of xerostomia. This limitation hinders a comprehensive understanding of the impact of xerostomia on quality of life in OLP patients [10]. Additionally, two studies examined the relationship between fungal microflora and xerostomia in OLP patients. One study reported a significant prevalence of *Candida* spp. in OLP lesions [3], while the other found no correlation between *Candida* colonization and dry mouth [4]. These conflicting results could be due to differences in sampling methods and the lack of consideration for the clinical subtypes of OLP lesions. Future research should investigate whether specific clinical forms of OLP, such as erosive or atrophic types, are more prone to fungal colonization and xerostomia, which could provide insights into targeted management strategies.

A study on total antioxidant status in OLP patients indicated that reduced antioxidant levels may exacerbate xerostomia, suggesting a potential therapeutic role for antioxidant supplementation. However, the study did not establish clear cut‐off values for what constitutes a clinically significant reduction in antioxidants, nor did it differentiate the impact of aging on salivary antioxidant levels [5]. Lastly, The role of muscarinic acetylcholine receptors (mAChR), particularly the M3 subtype, in salivary secretion was also highlighted [11]. While studies suggest that M3 receptor dysfunction may contribute to xerostomia in OLP [6], the mechanisms remain speculative. The hypothesis that OLP may involve an autoimmune process affecting M3 receptors through antibody production has not been conclusively proven, as OLP is classified as a type 4 immuno‐inflammatory disorder rather than a classical autoimmune disease [82]. The most recent WHO 2020 diagnostic criteria for OLP [83], which supersede the 2016 AAOMP criteria [84], emphasize the immuno‐inflammatory nature of the disorder, reinforcing the need for more research into its underlying pathophysiology. The papers on xerostomia in patients with Sjogren's syndrome, the importance of agonists of the M3 receptor itself, antibodies against the M3 receptor, and antagonists of the M3 receptor have been examined thoroughly [12, 13]. In OLP patients, it can be based on the hypothesis that xerostomia through three mechanisms including activation of M3 antagonists leads to blockade of the M3 receptor, thereby inhibiting the typical binding of acetylcholine agonist to its receptor, or the mechanism of antibody against M3 receptor, which due to activation of its cells degrades immunity against its receptor. This mechanism can be due to the autoimmune nature through humoral immunity in the disease. Furthermore, the mechanism of impairment in the intracellular Cascades such as calcium and K concentration is important in the transmission of the message resulting from the M3 receptor, all of which may be involved in the incidence of sense of dry mouth in OLP patients.

The latest group of studies investigated had dealt with inspecting the sense of dry mouth in response to the M3 receptor, evaluating the existence of the sense of dry mouth as well as its severity along with investigating the M3 receptor of stimulated saliva, nonstimulated saliva, and the lower lip minor salivary glands by applying questionnaires concurrently. Its result was an association between xerostomia and the quantity of M3 receptors [14, 15]

It seems that future studies on a wider scale to investigate auto‐antibody against M3 concurrent with examining inflammatory factors of IgG1, IgG3, and IgA in the cause of OLP autoimmune and inflammatory disease can reveal the etiopathogenesis of dry mouth in these patients.

Mucin is a lubricant and softener factor, which protects the oral tissue and supports mastication, swallowing, and speaking. It has also antimicrobial properties and is known as the first line of defense for the epithelial layer, which has defensive and immunity tasks. In previous studies in this regard, the extent and role of mucin with regard to xerostomia in patients with dry mouth etiology such as Sjogren's and Graves's diseases have been examined. The last study of this group reported a significant relationship between stimulated hyposalivation and reduced levels of B5 mucin in the saliva of patients [1].

Overall, the current literature lacks standardized methodologies and comprehensive analyses that consider age‐related changes in salivary function, the clinical heterogeneity of OLP, and the impact of systemic factors on xerostomia. Future studies should adopt a more holistic approach, integrating immunological, biochemical, and clinical data to elucidate the complex interactions between OLP and xerostomia. Incorporating the latest WHO diagnostic criteria and assessing both the presence and severity of xerostomia in a standardized manner will enable more informative conclusions and potentially lead to better management strategies for OLP patients.

## 5 Conclusion

This systematic review has provided a comprehensive overview of the current literature on the relationship or co‐occurrence between OLP and xerostomia. While the review identified potential associations and highlighted several factors contributing to xerostomia in OLP patients, it also revealed significant gaps and inconsistencies in existing research. The variations in study methodologies, lack of standardized diagnostic criteria, and insufficient examination of confounding variables such as age and systemic health limit the generalizability of findings. Future research should address these limitations by focusing on the following key areas:

1. Different forms of OLP (erosive, atrophic, reticular).
2. Fungal infection regarding the quantity and quality of the severity of inflammation resulting from different forms of OLP.
3. The effect of antibodies on receptors.
4. The effect of antagonists on receptors.
5. Investigating blood factors including IgA, IgG1, and IgG3 for examining inflammation.
6. The effect of effective neuropeptides on secretion such as substance p, intestinal peptide vasoactive, calcium, etc. in saliva.

Addressing these aspects will enable a more precise understanding of the etiopathogenesis of xerostomia in OLP patients, including the involvement of the central nervous system, autonomic nervous system, receptor pathways, cytokine interactions, and water transport mechanisms. Such insights will be crucial for developing targeted diagnostic and therapeutic strategies aimed at improving the quality of life for OLP patients by effectively managing xerostomia. Overall, while this review offers a categorized synthesis of recent data on OLP and xerostomia, it acknowledges the limitations posed by the small number of studies, challenges in standardizing inclusion criteria, potential selection bias, and the complexities in data interpretation. Future research, with a more rigorous and structured approach, will be instrumental in addressing these challenges, thereby advancing the understanding and management of xerostomia in OLP patients.

## Author Contributions

**Farzaneh AghaHosseini:** conceptualization, methodology, project administration, supervision, validation, visualization, writing–original draft, writing–review and editing. **Maryam Tahmasebinasab:** investigation, methodology, software, writing–original draft, writing–review and editing. **Mehdi Vatanpour:** methodology, visualization, writing–original draft, writing–review and editing.

## Consent

Informed consent was obtained from the patient for the publication of this case report and accompanying images.

## Conflicts of Interest

The authors declare no conflicts of interest.

## Data Availability

The data that support the findings of this study are available on request from the corresponding author.

## References

[1] F. Agha‐Hosseini , M. Imanpour , I. Mirzaii‐Dizgah , and M. S. Moosavi , “Mucin 5B in Saliva and Serum of Patients With Oral Lichen Planus,” Scientific Reports 7, no. 1 (2017): 12060, 10.1038/s41598-017-12157-1. 28935947 PMC5608939

[2] K. R. Larsen , J. D. Johansen , J. Reibel , C. Zachariae , K. Rosing , and A. M. L. Pedersen , “Oral Symptoms and Salivary Findings in Oral Lichen Planus, Oral Lichenoid Lesions, and Stomatitis,” BMC Oral Health 17, no. 1 (2017): 103, 10.1186/s12903-017-0393-2. 28662707 PMC5492674

[3] M. Bokor‐Bratic , M. Jankovic , and N. Dragnic , “Unstimulated Whole Salivary Flow Rate and Anxiolytic Intake Are Independently Associated With Oral Candida Infection in Patients With Oral Lichen Planus,” European Journal of Oral Sciences 121, no. 5 (2013): 427–433. 24028590 10.1111/eos.12073

[4] G. Artico , R. Freitas , A. Santos Filho , G. Benard , R. Romiti , and D. Migliari , “Prevalence of Candida Spp., Xerostomia, and Hyposalivation in Oral Lichen Planus—A Controlled Study,” Oral Diseases 20, no. 3 (2014): e36–e41, 10.1111/odi.12120. 23656547

[5] A. Tvarijonaviciute , C. Aznar‐Cayuela , C. P. Rubio , F. Tecles , J. J. Ceron , and P. López‐Jornet , “Salivary Antioxidant Status in Patients With Oral Lichen Planus: Correlation With Clinical Signs and Evolution During Treatment With *Chamaemelum nobile* ,” BioMed Research International 2018 (2018): 1–5, 10.1155/2018/5187549. PMC5994308 29992150

[6] P. Abrams , K. E. Andersson , J. J. Buccafusco , et al., “Muscarinic Receptors: Their Distribution and Function in Body Systems, and the Implications for Treating Overactive Bladder,” British Journal of Pharmacology 148, no. 5 (2006): 565–578, 10.1038/sj.bjp.0706780. 16751797 PMC1751864

[7] C. R. Fluixa , J. V. Bagan Sebastian , M. A. Milian Masanet , Y. Jimenez Soriano , and E. Lloria de Miguel , “Quantitative Analysis of Saliva in Patients With Oral Lichen Planus: A Study of 100 Cases,” Medicina Oral 5, no. 3 (2000): 187–192. 11507555

[8] A. N. Colquhoun , An Investigation of the Association Between Oral Lichen Planus and a Persistently Dry Mouth: A Research Report Submitted in Partial Fulfilment of the Requirements for the Degree of Master of Dental Surgery (Oral and Maxillofacial Surgery) of the University of Otago (Dunedin, New Zealand: University of Otago, 2002).

[9] B. K. Gandara , K. T. Izutsu , E. L. Truelove , I. D. Mandel , E. E. Sommers , and W. Y. Ensign , “Sialochemistry of Whole, Parotid, and Labial Minor Gland Saliva in Patients With Oral Lichen Planus,” Journal of Dental Research 66, no. 11 (1987): 1619–1622, 10.1177/00220345870660110201. 10872393

[10] A. N. Colquhoun and M. M. Ferguson , “An Association Between Oral Lichen Planus and a Persistently Dry Mouth,” Oral Surgery, Oral Medicine, Oral Pathology, Oral Radiology, and Endodontology 98, no. 1 (2004): 60–68, 10.1016/j.tripleo.2003.11.003. 15243472

[11] T. Nakamura , M. Matsui , K. Uchida , et al., “M(3) Muscarinic Acetylcholine Receptor Plays a Critical Role in Parasympathetic Control of Salivation in Mice,” The Journal of Physiology 558, no. pt. 2 (2004): 561–575, 10.1113/jphysiol.2004.064626. 15146045 PMC1664962

[12] H. Tsuboi , M. Iizuka , H. Tsushima , and T. Sumida , “Anti‐M3 Muscarinic Acetylcholine Receptor Antibodies and Sjögren's Syndrome,” Nihon Rinsho Meneki Gakkai Kaishi 36, no. 2 (2013): 77–85, 10.2177/jsci.36.77. 23629427

[13] M. Tahara , H. Tsuboi , S. Segawa , et al., “RORγt Antagonist Suppresses M3 Muscarinic Acetylcholine Receptor‐Induced Sjögren's Syndrome‐Like Sialadenitis,” Clinical and Experimental Immunology 187, no. 2 (2017): 213–224, 10.1111/cei.12868. 27643385 PMC5217906

[14] F. Agha‐Hosseini , I. Mirzaii‐Dizgah , and N. Mohammadpour , “Muscarinic Cholinergic Receptors (MR3) in Saliva of Patients With Oral Lichen Planus,” Archives of Dermatological Research 308, no. 7 (2016): 481–486, 10.1007/s00403-016-1670-7. 27371099

[15] F. Agha‐Hosseini , M. S. Moosavi , I. Mirzaii‐Dizgah , and M. Samami , “Muscarinic Cholinergic Receptors in Minor Salivary Gland Tissues of Patients With Oral Lichen Planus: A Case‐Control Study,” Journal of Oral Pathology & Medicine 49, no. 8 (2020): 816–821, 10.1111/jop.13094. 32744338

[16] K. Oikarinen , T. Salo , M. Kylmäniemi , R. Palatsi , T. Karhunen , and A. Oikarinen , “Systemic Oral Isotretinoin Therapy and Flow Rate, pH, and Matrix Metalloproteinase‐9 Activity of Stimulated Saliva,” Acta Odontologica Scandinavica 53, no. 6 (1995): 369–371, 10.3109/00016359509006003. 8849870

[17] H. S. Kho , J. Y. Chang , Y. Y. Kim , and Y. Kim , “MUC1 and Toll‐Like Receptor‐2 Expression in Burning Mouth Syndrome and Oral Lichen Planus,” Archives of Oral Biology 58, no. 7 (2013): 837–842, 10.1016/j.archoralbio.2013.01.008. 23411403

[18] F. Agha‐Hosseini and M. S. Moosavi , “An Evidence‐Based Review Literature About Risk Indicators and Management of Unknown‐Origin Xerostomia,” Journal of Dentistry 10, no. 3 (2013): 273–282. 25512755 PMC4264100

[19] M. Tanasiewicz , T. Hildebrandt , and I. Obersztyn , “Xerostomia of Various Etiologies: A Review of the Literature,” Advances in Clinical and Experimental Medicine 25, no. 1 (2016): 199–206, 10.17219/acem/29375. 26935515

[20] A. M. Frydrych , “Dry Mouth: Xerostomia and Salivary Gland Hypofunction,” Australian Family Physician 45, no. 7 (2016): 488–492, https://search.informit.org/doi/abs/10.3316/informit.241742613329484. 27610431

[21] A. Tvarijonaviciute , C. Aznar‐Cayuela , C. P. Rubio , J. J. Ceron , and P. López‐Jornet , “Evaluation of Salivary Oxidate Stress Biomarkers, Nitric Oxide, and C‐Reactive Protein in Patients With Oral Lichen Planus and Burning Mouth Syndrome,” Journal of Oral Pathology & Medicine 46, no. 5 (2017): 387–392, 10.1111/jop.12522. 27862315

[22] A. Mansourian , N. Shanbehzadeh , S. J. Kia , and M. S. Moosavi , “Increased Salivary Aldehyde Dehydrogenase 1 in Non‐Reticular Oral Lichen Planus,” Anais Brasileiros de Dermatologia 92, no. 2 (2017): 168–171, 10.1590/abd1806-4841.20174964. 28538873 PMC5429099

[23] M.‐S. Moosavi and H. Barati , “Comment on ‘Xerostomia and Salivary Gland Hypofunction in Patients With Oral Lichen Planus Before and After Treatment With Topical Corticosteroids’,” The Open Dentistry Journal 12 (2018): 310–311. 29760823 10.2174/1874210601812010310 PMC5944121

[24] A. M. Frydrych , “Response to Comment on ‘Xerostomia and Salivary Gland Hypofunction in Patients With Oral Lichen Planus Before and After Treatment With Topical Corticosteroids’,” The Open Dentistry Journal 12, no. 1 (2018): 974–976, 10.2174/1874210601812010974. PMC5944121 29760823

[25] A. Mansourian , S. Najafi , N. Nojoumi , P. Parhami , and M. S. Moosavi , “Salivary Cortisol and Salivary Flow Rate in Clinical Types of Oral Lichen Planus,” Skinmed 16, no. 1 (2018): 19–22, https://europepmc.org/article/med/29551106. 29551106

[26] F. Agha‐Hosseini , I. Mirzaii‐Dizgah , M. Mohebbian , and M. R. Sarookani , “Vascular Endothelial Growth Factor in Serum and Saliva of Oral Lichen Planus and Oral Squamous Cell Carcinoma Patients,” Journal of Kerman University of Medical Sciences 25, no. 1 (2018): 27–33, https://jkmu.kmu.ac.ir/article_63173.htmlhttps://jkmu.kmu.ac.ir/article_63173.html.

[27] M. H. Mirzaii‐Dizgah , B. Rohani , and I. Mirzaii‐Dizgah , “Complements C3 and C4 in Serum and Stimulatedsaliva of Patients Suffer Oral Erosive Lichen Planus,” Physiology and Pharmacology 25, no. 2 (2021): 102–107, 10.32598/ppj.25.2.50.

[28] N. Sheykhbahaei and N. Gholizadeh , “Serum and Salivary Levels of Vitamin D in Oral Lichen Planus Patients,” Koomesh Journal 23, no. 3 (2021): 379–385, 10.29252/koomesh.23.3.379.

[29] S. Yiemstan , S. Krisdapong , and P. Piboonratanakit , “Association Between Clinical Signs of Oral Lichen Planus and Oral Health‐Related Quality of Life: A Preliminary Study,” Dentistry Journal 8, no. 4 (2020): 113, 10.3390/dj8040113. 33020378 PMC7711772

[30] A. Osipoff , M. D. Carpenter , J. L. Noll , J. A. Valdez , M. Gormsen , and M. T. Brennan , “Predictors of Symptomatic Oral Lichen Planus,” Oral Surgery, Oral Medicine, Oral Pathology and Oral Radiology 129, no. 5 (2020): 468–477, 10.1016/j.oooo.2019.12.019. 32044267

[31] H. Çevik‐Aras , S. Musa , R. Olofsson , A. Almståhl , and U. Almhöjd , “Patients With Oral Lichen Planus Display Lower Levels of Salivary Acidic Glycoproteins Than Individuals Without Oral Mucosal Disease,” Clinical Oral Investigations 28, no. 1 (2023): 2. 38114810 10.1007/s00784-023-05411-6 PMC10730629

[32] V. Bouvard , S. T. Nethan , D. Singh , et al., “IARC Perspective on Oral Cancer Prevention,” New England Journal of Medicine 387, no. 21 (November 2022): 1999–2005, 10.1056/NEJMsr2210097. 36378601

[33] A. Salem , A. Al‐Samadi , V. Stegajev , et al., “Histamine H4 Receptor in Oral Lichen Planus,” Oral Diseases 21, no. 3 (April 2015): 378–385, 10.1111/odi.12290. 25207698

[34] E. Shavit , K. Hagen , and N. Shear , “Oral Lichen Planus: A Novel Staging and Algorithmic Approach and All That Is Essential to Know,” F1000Research 9 (2020): 206, 10.12688/f1000research.18713.1. PMC7096219 32226613

[35] M. A. Hashemipour , S. Sheikhhoseini , Z. Afshari , and A. R. Gandjalikhan Nassab , “The Relationship Between Clinical Symptoms of Oral Lichen Planus and Quality of Life Related to Oral Health,” BMC Oral Health 24, no. 1 (May 2024): 556. 38735922 10.1186/s12903-024-04326-2 PMC11089796

[36] K. R. Larsen , J. D. Johansen , J. Reibel , C. Zachariae , K. Rosing , and A. M. L. Pedersen , “Oral Symptoms and Salivary Findings in Oral Lichen Planus, Oral Lichenoid Lesions and Stomatitis,” BMC Oral Health 17 (2017): 103, 10.1186/s12903-017-0393-2. 28662707 PMC5492674

[37] M. Baharvand , A. Khodadoustan , M. Mohammadi , H. Mortazavi , and A. Movahhedian , “Xerostomia Due to Systemic Disease: A Review of 20 Conditions and Mechanisms,” Annals of Medical and Health Sciences Research 4, no. 4 (2014): 503–510, 10.4103/2141-9248.139284. 25221694 PMC4160670

[38] M. Bokor‐Bratic , M. Cankovic , and N. Dragnic , “Unstimulated Whole Salivary Flow Rate and Anxiolytics Intake Are Independently Associated With Oral C Andida Infection in Patients With Oral Lichen Planus,” European Journal of Oral Sciences 121, no. 5 (October 2013): 427–433, 10.1111/eos.12073. 24028590

[39] B. K. Gandara , K. T. Izutsu , E. L. Truelove , I. D. Mandel , E. E. Sommers , and W. Y. Ensign , “Sialochemistry of Whole, Parotid, and Labial Minor Gland Saliva in Patients With Oral Lichen Planus,” Journal of Dental Research 66, no. 11 (November 1987): 1619–1622, 10.1177/00220345870660110201. 10872393

[40] A. N. Colquhoun and M. M. Ferguson , “An Association Between Oral Lichen Planus and a Persistently Dry Mouth,” Oral Surgery, Oral Medicine, Oral Pathology, Oral Radiology, and Endodontology 98, no. 1 (July 2004): 60–68, 10.1016/j.tripleo.2003.11.003. 15243472

[41] I. C. Lundström , K. Göran , B. Anneroth , and H. F. Bergstedt , “Salivary Gland Function and Changes in Patients With Oral Lichen Planus,” European Journal of Oral Sciences 90, no. 6 (December 1982): 443–458, 10.1111/j.1600-0722.1982.tb00761.x. 6961510

[42] A. Tvarijonaviciute , C. Aznar‐Cayuela , C. P. Rubio , F. Tecles , J. J. Ceron , and P. López‐Jornet , “Salivary Antioxidant Status in Patients With Oral Lichen Planus: Correlation With Clinical Signs and Evolution During Treatment With *Chamaemelum nobile* ,” BioMed Research International 2018, no. 1 (2018): 5187549, 10.1155/2018/5187549. 29992150 PMC5994308

[43] B. K. Gandara , K. T. Izutsu , E. L. Truelove , I. D. Mandel , E. E. Sommers , and W. Y. Ensign , “Sialochemistry of Whole, Parotid, and Labial Minor Gland Saliva in Patients With Oral Lichen Planus,” Journal of Dental Research 66, no. 11 (November 1987): 1619–1622, 10.1177/00220345870660110201. 10872393

[44] A. N. Colquhoun and M. M. Ferguson , “An Association Between Oral Lichen Planus and a Persistently Dry Mouth,” Oral Surgery, Oral Medicine, Oral Pathology, Oral Radiology, and Endodontology 98, no. 1 (July 2004): 60–68, 10.1016/j.tripleo.2003.11.003. 15243472

[45] M. Bokor‐Bratic , M. Cankovic , and N. Dragnic , “Unstimulated Whole Salivary Flow Rate and Anxiolytics Intake Are Independently Associated With Oral C Andida Infection in Patients With Oral Lichen Planus,” European Journal of Oral Sciences 121, no. 5 (October 2013): 427–433. 24028590 10.1111/eos.12073

[46] G. Artico , R. Freitas , A. Santos Filho , G. Benard , R. Romiti , and D. Migliari , “Prevalence of C Andida Spp., Xerostomia, and Hyposalivation in Oral Lichen Planus–A Controlled Study,” Oral Diseases 20, no. 3 (April 2014): e36–e41. 23656547 10.1111/odi.12120

[47] F. Agha‐Hosseini , I. Mirzaii‐Dizgah , and N. Mohammadpour , “Muscarinic Cholinergic Receptors (MR3) in Saliva of Patients With Oral Lichen Planus,” Archives of Dermatological Research 308 (September 2016): 481–486, 10.1007/s00403-016-1670-7. 27371099

[48] K. R. Larsen , J. D. Johansen , J. Reibel , C. Zachariae , K. Rosing , and A. M. L. Pedersen , “Oral Symptoms and Salivary Findings in Oral Lichen Planus, Oral Lichenoid Lesions and Stomatitis,” BMC Oral Health 17 (December 2017): 103, 10.1186/s12903-017-0393-2. 28662707 PMC5492674

[49] F. Agha‐Hosseini , M. Imanpour , I. Mirzaii‐Dizgah , and M. S. Moosavi , “Mucin 5B in Saliva and Serum of Patients With Oral Lichen Planus,” Scientific Reports 7, no. 1 (2017): 12060. 28935947 10.1038/s41598-017-12157-1 PMC5608939

[50] F. Agha‐Hosseini , M. S. Moosavi , I. Mirzaii‐Dizgah , and M. Samami , “Muscarinic Cholinergic Receptors in Minor Salivary Gland Tissues of Patients With Oral Lichen Planus: A Case‐Control Study,” Journal of Oral Pathology & Medicine: Official Publication of the International Association of Oral Pathologists and the American Academy of Oral Pathology 49, no. 8 (September 2020): 816–821, 10.1111/jop.13094. 32744338

[51] A. Tvarijonaviciute , C. Aznar‐Cayuela , C. P. Rubio , F. Tecles , J. J. Ceron , and P. López‐Jornet , “Salivary Antioxidant Status in Patients With Oral Lichen Planus: Correlation With Clinical Signs and Evolution During Treatment With *Chamaemelum nobile* ,” BioMed Research International 2018, no. 1 (2018): 5187549, 10.1155/2018/5187549. 29992150 PMC5994308

[52] B. K. Gandara , K. T. Izutsu , E. L. Truelove , I. D. Mandel , E. E. Sommers , and W. Y. Ensign , “Sialochemistry of Whole, Parotid, and Labial Minor Gland Saliva in Patients With Oral Lichen Planus,” Journal of Dental Research 66, no. 11 (November 1987): 1619–1622, 10.1177/00220345870660110201. 10872393

[53] A. N. Colquhoun and M. M. Ferguson , “An Association Between Oral Lichen Planus and a Persistently Dry Mouth,” Oral Surgery, Oral Medicine, Oral Pathology, Oral Radiology, and Endodontology 98, no. 1 (July 2004): 60–68, 10.1016/j.tripleo.2003.11.003. 15243472

[54] M. Bokor‐Bratic , M. Cankovic , and N. Dragnic , “Unstimulated Whole Salivary Flow Rate and Anxiolytics Intake Are Independently Associated With Oral C Andida Infection in Patients With Oral Lichen Planus,” European Journal of Oral Sciences 121, no. 5 (October 2013): 427–433, 10.1111/eos.12073. 24028590

[55] G. Artico , R. Freitas , A. Santos Filho , G. Benard , R. Romiti , and D. Migliari , “Prevalence of C Andida Spp., Xerostomia, and Hyposalivation in Oral Lichen Planus–A Controlled Study,” Oral Diseases 20, no. 3 (April 2014): e36–e41. 23656547 10.1111/odi.12120

[56] F. Agha‐Hosseini , I. Mirzaii‐Dizgah , and N. Mohammadpour , “Muscarinic Cholinergic Receptors (MR3) in Saliva of Patients With Oral Lichen Planus,” Archives of Dermatological Research 308 (September 2016): 481–486, 10.1007/s00403-016-1670-7. 27371099

[57] K. R. Larsen , J. D. Johansen , J. Reibel , C. Zachariae , K. Rosing , and A. M. L. Pedersen , “Oral Symptoms and Salivary Findings in Oral Lichen Planus, Oral Lichenoid Lesions and Stomatitis,” BMC Oral health 17 (December 2017): 103, 10.1186/s12903-017-0393-2. 28662707 PMC5492674

[58] F. Agha‐Hosseini , M. Imanpour , I. Mirzaii‐Dizgah , and M. S. Moosavi , “Mucin 5B in Saliva and Serum of Patients With Oral Lichen Planus,” Scientific Reports 7, no. 1 (September 2017): 12060. 28935947 10.1038/s41598-017-12157-1 PMC5608939

[59] F. Agha‐Hosseini , M. S. Moosavi , I. Mirzaii‐Dizgah , and M. Samami , “Muscarinic Cholinergic Receptors in Minor Salivary Gland Tissues of Patients With Oral Lichen Planus: A Case‐Control Study,” Journal of Oral Pathology & Medicine: Official Publication of the International Association of Oral Pathologists and the American Academy of Oral Pathology 49, no. 8 (September 2020): 816–821, 10.1111/jop.13094. 32744338

[60] F. Agha‐Hosseini , I. Mirzaii‐Dizgah , and N. Mohammadpour , “Muscarinic Cholinergic Receptors (MR3) in Saliva of Patients With Oral Lichen Planus,” Archives of Dermatological Research 308 (September 2016): 481–486. 27371099 10.1007/s00403-016-1670-7

[61] A. N. Colquhoun and M. M. Ferguson , “An Association Between Oral Lichen Planus and a Persistently Dry Mouth,” Oral Surgery, Oral Medicine, Oral Pathology, Oral Radiology, and Endodontology 98, no. 1 (July 2004): 60–68. 10.1016/j.tripleo.2003.11.003 15243472

[62] M. Bokor‐Bratic , M. Cankovic , and N. Dragnic , “Unstimulated Whole Salivary Flow Rate and Anxiolytics Intake Are Independently Associated With Oral C Andida Infection in Patients With Oral Lichen Planus,” European Journal of Oral Sciences 121, no. 5 (October 2013): 427–433. 24028590 10.1111/eos.12073

[63] K. R. Larsen , J. D. Johansen , J. Reibel , C. Zachariae , K. Rosing , and A. M. L. Pedersen , “Oral Symptoms and Salivary Findings in Oral Lichen Planus, Oral Lichenoid Lesions and Stomatitis,” BMC Oral Health 17 (2017): 103. 28662707 10.1186/s12903-017-0393-2 PMC5492674

[64] G. Artico , R. Freitas , A. Santos Filho , G. Benard , R. Romiti , and D. Migliari , “Prevalence of C Andida Spp., Xerostomia, and Hyposalivation in Oral Lichen Planus—A Controlled Study,” Oral Diseases 20, no. 3 (April 2014): e36–e41. 23656547 10.1111/odi.12120

[65] A. Tvarijonaviciute , C. Aznar‐Cayuela , C. P. Rubio , F. Tecles , J. J. Ceron , and P. López‐Jornet , “Salivary Antioxidant Status in Patients With Oral Lichen Planus: Correlation With Clinical Signs and Evolution During Treatment With *Chamaemelum nobile* ,” BioMed Research International 2018, no. 1 (2018): 1–5. 10.1155/2018/5187549 PMC5994308 29992150

[66] B. K. Gandara , K. T. Izutsu , E. L. Truelove , I. D. Mandel , E. E. Sommers , and W. Y. Ensign , “Sialochemistry of Whole, Parotid, and Labial Minor Gland Saliva in Patients With Oral Lichen Planus,” Journal of Dental Research 66, no. 11 (November 1987): 1619–1622. 10872393 10.1177/00220345870660110201

[67] A. N. Colquhoun and M. M. Ferguson , “An Association Between Oral Lichen Planus and a Persistently Dry Mouth,” Oral Surgery, Oral Medicine, Oral Pathology, Oral Radiology, and Endodontology 98, no. 1 (July 2004): 60–68, 10.1016/j.tripleo.2003.11.003. 15243472

[68] A. Tvarijonaviciute , C. Aznar‐Cayuela , C. P. Rubio , F. Tecles , J. J. Ceron , and P. López‐Jornet , “Salivary Antioxidant Status in Patients With Oral Lichen Planus: Correlation With Clinical Signs and Evolution During Treatment With *Chamaemelum nobile* ,” BioMed Research International 2018, no. 1 (2018): 5187549, 10.1155/2018/5187549. 29992150 PMC5994308

[69] M. Bokor‐Bratic , M. Cankovic , and N. Dragnic , “Unstimulated Whole Salivary Flow Rate and Anxiolytics Intake Are Independently Associated With Oral C Andida Infection in Patients With Oral Lichen Planus,” European Journal of Oral Sciences 121, no. 5 (October 2013): 427–433. 24028590 10.1111/eos.12073

[70] B. K. Gandara , K. T. Izutsu , E. L. Truelove , I. D. Mandel , E. E. Sommers , and W. Y. Ensign , “Sialochemistry of Whole, Parotid, and Labial Minor Gland Saliva in Patients With Oral Lichen Planus,” Journal of Dental Research 66, no. 11 (November 1987): 1619–1622, 10.1177/00220345870660110201. 10872393

[71] K. R. Larsen , J. D. Johansen , J. Reibel , C. Zachariae , K. Rosing , and A. M. L. Pedersen , “Oral Symptoms and Salivary Findings in Oral Lichen Planus, Oral Lichenoid Lesions and Stomatitis,” BMC Oral Health 17 (December 2017): 103. 28662707 10.1186/s12903-017-0393-2 PMC5492674

[72] A. N. Colquhoun and M. M. Ferguson , “An Association Between Oral Lichen Planus and a Persistently Dry Mouth,” Oral Surgery, Oral Medicine, Oral Pathology, Oral Radiology, and Endodontology 98, no. 1 (July 2004): 60–68. 10.1016/j.tripleo.2003.11.003 15243472

[73] G. Artico , R. Freitas , A. Santos Filho , G. Benard , R. Romiti , and D. Migliari , “Prevalence of C Andida Spp., Xerostomia, and Hyposalivation in Oral Lichen Planus—A Controlled Study,” Oral Diseases 20, no. 3 (April 2014): e36–e41. 23656547 10.1111/odi.12120

[74] A. Tvarijonaviciute , C. Aznar‐Cayuela , C. P. Rubio , F. Tecles , J. J. Ceron , and P. López‐Jornet , “Salivary Antioxidant Status in Patients With Oral Lichen Planus: Correlation With Clinical Signs and Evolution During Treatment With *Chamaemelum nobile* ,” BioMed Research International 2018, no. 1 (2018): 1–5. 10.1155/2018/5187549 PMC5994308 29992150

[75] F. Agha‐Hosseini , I. Mirzaii‐Dizgah , and N. Mohammadpour , “Muscarinic Cholinergic Receptors (MR3) in Saliva of Patients With Oral Lichen Planus,” Archives of Dermatological Research 308 (September 2016): 481–486, 10.1007/s00403-016-1670-7. 27371099

[76] K. R. Larsen , J. D. Johansen , J. Reibel , C. Zachariae , K. Rosing , and A. M. L. Pedersen , “Oral Symptoms and Salivary Findings in Oral Lichen Planus, Oral Lichenoid Lesions and Stomatitis,” BMC Oral health 17 (2017): 103. 28662707 10.1186/s12903-017-0393-2 PMC5492674

[77] F. Agha‐Hosseini , M. Imanpour , I. Mirzaii‐Dizgah , and M. S. Moosavi , “Mucin 5B in Saliva and Serum of Patients With Oral Lichen Planus,” Scientific Reports 7, no. 1 (September 2017): 12060. 28935947 10.1038/s41598-017-12157-1 PMC5608939

[78] K. R. Larsen , J. D. Johansen , J. Reibel , C. Zachariae , K. Rosing , and A. M. L. Pedersen , “Oral Symptoms and Salivary Findings in Oral Lichen Planus, Oral Lichenoid Lesions and Stomatitis,” BMC Oral Health 17 (December 2017): 103. 28662707 10.1186/s12903-017-0393-2 PMC5492674

[79] F. Agha‐Hosseini , M. Imanpour , I. Mirzaii‐Dizgah , and M. S. Moosavi , “Mucin 5B in Saliva and Serum of Patients With Oral Lichen Planus,” Scientific Reports 7, no. 1 (September 2017): 12060. 28935947 10.1038/s41598-017-12157-1 PMC5608939

[80] F. Agha‐Hosseini , I. Mirzaii‐Dizgah , and N. Mohammadpour , “Muscarinic Cholinergic Receptors (MR3) in Saliva of Patients With Oral Lichen Planus,” Archives of Dermatological Research 308 (September 2016): 481–486. 27371099 10.1007/s00403-016-1670-7

[81] F. Agha‐Hosseini , M. S. Moosavi , I. Mirzaii‐Dizgah , and M. Samami , “Muscarinic Cholinergic Receptors in Minor Salivary Gland Tissues of Patients With Oral Lichen Planus: A Case‐Control Study,” Journal of Oral Pathology & Medicine: Official publication of the International Association of Oral Pathologists and the American Academy of Oral Pathology 49, no. 8 (September 2020): 816–821. 32744338 10.1111/jop.13094

[82] J. Wang , J. Yang , W. Xia , et al., “Escherichia Coli Enhances Th17/Treg Imbalance via TLR4/NF‐κB Signaling Pathway in Oral Lichen Planus,” International Immunopharmacology 119 (June 2023): 110175. 37058754 10.1016/j.intimp.2023.110175

[83] D. I. Rotaru , D. Sofineti , S. D Bolboacă , and A. E Bulboacă , “Dijagnostički Kriteriji Za Oralni Lihen Planus: Narativni Pregled,” Acta Clinica Croatica 59, no. 3 (September 2020): 513–522. 34177062 10.20471/acc.2020.59.03.16 PMC8212651

[84] Y. S. L. Cheng , A. Gould , Z. Kurago , J. Fantasia , and S. Muller , “Diagnosis of Oral Lichen Planus: A Position Paper of the American Academy of Oral and Maxillofacial Pathology,” Oral Surgery, Oral Medicine, Oral Pathology and Oral Radiology 122, no. 3 (September 2016): 332–354. 27401683 10.1016/j.oooo.2016.05.004

[85] I. C. Lundström , K. Göran , B. Anneroth , and H. F. Bergstedt , “Salivary Gland Function and Changes in Patients With Oral Lichen Planus,” European Journal of Oral Sciences 90, no. 6 (December 1982): 443–458, 10.1111/j.1600-0722.1982.tb00761.x. 6961510

[86] H. Al‐Janaby , H. El‐Sakka , M. Masood , et al., “Xerostomia and Salivary Gland Hypofunction in Patients With Oral Lichen Planus Before and After Treatment With Topical Corticosteroids,” The Open Dentistry Journal 11 (2017): 155–163, 10.2174/1874210601711010155. 28567139 PMC5418946

[87] M. H. Edens , M. D. Carpenter , J. J. Napeñas , and M. T. Brennan , “Impact of Salivary Hypofunction on Incidence of Orofungal Infections With Use of Topical Steroids for Management of Oral Lichen Planus and Xerostomia,” Oral Surgery, Oral Medicine, Oral Pathology and Oral Radiology 126, no. 6 (December 2018): 501–505, 10.1016/j.oooo.2018.06.012. 30309830

[88] F. Agha‐Hosseini , M. S. Moosavi , I. Mirzaii‐Dizgah , and M. Samami , “Muscarinic Cholinergic Receptors in Minor Salivary Gland Tissues of Patients With Oral Lichen Planus: A Case‐Control Study,” Journal of Oral Pathology & Medicine: Official Publication of the International Association of Oral Pathologists and the American Academy of Oral Pathology 49, no. 8 (September 2020): 816–821, 10.1111/jop.13094. 32744338

